# Women’s knowledge, attitudes and practices (KAP) relating to breast and cervical cancers in rural Zimbabwe: a cross sectional study in Mudzi District, Mashonaland East Province

**DOI:** 10.1186/s12889-018-6333-5

**Published:** 2019-01-24

**Authors:** Lovemore Makurirofa, Priscilla Mangwiro, Victoria James, Amos Milanzi, Junior Mavu, Michael Nyamuranga, Sydney Kamtauni

**Affiliations:** 1 Cancer Association of Zimbabwe, Harare, 60 Livingstone Avenue Conner 6th Street, Box 3358, Zimbabwe; 2 Allied Health Practitioners Council of Zimbabwe, Harare, Zimbabwe; 3 New Dimension Consultancy, Harare, Zimbabwe; 4 Nurses Council of Zimbabwe, Harare, Zimbabwe; 5 Ministry of Health and Child Care, Mudzi district, Zimbabwe; 6 Cancer Association of Zimbabwe, Mudzi district, Zimbabwe

**Keywords:** Breast cancer, Cervical cancer, Cross-sectional study, Knowledge, Attitudes, And practices, Women, Zimbabwe

## Abstract

**Background:**

Breast and cervical cancers constitute the most common cancers among women in sub-Saharan Africa. In Zimbabwe, cervical cancer accounts for more than a third of all cancers among women of African descent. Cancer knowledge levels, attitudes and practices of people in different sections of society should be assessed in order to guide current cancer interventions. This study aimed to assess breast and cervical cancer knowledge, attitudes and practices of women of reproductive age, in Mudzi District, Republic of Zimbabwe.

**Methods:**

A cross-sectional community-based survey was conducted. A total of 409 survey household questionnaires were administered to women of reproductive age (15–49 years) in 2014.

**Results:**

A total of 409 respondents were interviewed. Nearly 85% of respondents had heard of cancer. 34.2% did not know of any cervical cancer risk factors and 51% were not familiar with the signs and symptoms of cervical cancer. Fifty five percent (55%) had not discussed cancer issues with partners in the past 12 months, and only 27.4% had discussed cancer issues with partners at all. Most of the respondents (96.2%) had never undergone cervical cancer screening. The majority of the respondents (70.8%) had never discussed breast cancer issues with community members. About 70% had never discussed cervical cancer issues with community members.

**Conclusions:**

This study revealed a lack of awareness and comprehensive knowledge about breast and cervical cancer. It also revealed low self-risk perception, low uptake of cancer early detection services and low capacity of the local health institution in offering cancer services. It is recommended that the scaling-up of cancer information, dissemination, and early detection services must be prioritised, including training of local health institutions.

## Background

Breast and cervical cancers are the most common cancers among women in Sub-Saharan Africa (SSA) [1]. In Zimbabwe, over 5000 new cancer diagnoses and over 1000 cancer-related deaths are recorded every year [2]. The incidence of cancer in Zimbabwe is on the rise; it increased from 2728 new cases in 2008, to 7165 in 2015. Breast and cervical cancer constituted a respective 7 and 19% of Zimbabwe’s total cancer incidences in 2015. Breast and cervical cancer alone contributed 11.6 and 34.8%, respectively, to the total cancer incidences of indigenous Zimbabwean women in 2015. Breast and cervical cancer constituted 7 and 12%, respectively, of the total number of cancer deaths recorded in 2015 [3]. Despite the increase in cervical and breast cancer cases, current cancer screening coverage and accessibility to screening services in Zimbabwe is still limited. The estimated cervical cancer screening coverage among the 15 to 49 age group in 2015 was 13%. Urban - rural disparities still exist (21% in urban as compared to 7% in rural áreas) [4, 5].

The above figures likely underestimate the magnitude of the overall cancer burden in Zimbabwe because of centralisation of the cancer registry [2]. Similarly to the situation in other sub-Saharan African countries, the majority of cancer cases are detected very late. This generally results in poor prognosis. An estimated 84% of the staged cancer patients in Zimbabwe (29%) present with advanced stages of the disease [3]. Some of the many possible reasons for late detection include common myths and misconception about cancer, and lack of access to early detection services. A study in Bindura District, Zimbabwe, for example, revealed that the majority of respondents had not heard of breast self-examination and that more than half did not think that they could develop breast cancer. The majority of the respondents demonstrated low levels of knowledge regarding breast and cervical cancer [6].

Studies elsewhere in sub-Saharan Africa show limited knowledge of cervical cancer among the general population. Lack of information and misinformation about cervical cancer have been documented in countries as disparate as Ethiopia, (Chaka B, Sayed A, Goeieman B, Rayne S: A survey of knowledge, attitudes, to cervical and bresat cancers among women in Ethiopia, submitted) Ghana, [7] South Africa, [8] Sudan [9] and Tanzania [10]. A study conducted among hospital staff in Mulago hospital in Uganda found that medical workers, who are ordinarily responsible for their patients’ cancer testing, do not necessarily undergo screening themselves. Over half of these respondents did not feel that they were susceptible to cervical cancer, and the majority had never been screened [11]. This study showed a low level of cervical cancer knowledge among medical staff. A study in Zimbabwe’s Mudzi District also revealed lack of knowledge and incorrect beliefs about the risk of developing cervical cancer among health workers. The majority of respondents did not know about human papilloma virus (HPV) screening, or about the visual inspection of the cervix using acetic acid. Most believed that they were not at risk of developing cervical cancer and most of the respondents (81.7%) had not undergone cervical cancer screening [12].

Only two previous studies have assessed breast and cervical knowledge in Zimbabwe [6, 12]. The study focused on breast cancer knowledge levels in an urban set up only [6] while the other earlier study focused on the knowledge of healthcare workers only [12]. The knowledge, attitudes and practices of women in Mudzi, however, have not been assessed. This study, therefore, aims to fill this literature gap and is the first KAP study done at the district level in an exclusively rural context in Zimbabwe. This study seeks to determine the breast and cervical cancer knowledge levels, attitudes and practices of women of reproductive age, in Mudzi District, Republic of Zimbabwe.

## Methods

A descriptive, cross-sectional study was carried out in Mudzi District, Zimbabwe among women between the ages of 15 and 49. The district is one of the most remote rural communities in Zimbabwe, at the border with Mozambique. It is approximately 250 km east of the capital, Harare.

The study employed quantitative research methods. A total of 409 survey household questionnaires were administered through interviews by pre-survey trained and qualified personnel to women of reproductive age (15–49 years old) in the five wards of the Mudzi District, in 2014. The study focused on women between 15 and 49 years because that was the target group of the cancer education and screening project which was incorporated in the existing HIV and AIDS interventions in Mudzi district which already targeted the reproductive age group (15–49 years age group). The questionnaire was pilot tested. The training of data collection staff and pilot testing of the data collection tool was done to minimise potentiation bias. Likert scale was employed in generating the attitudes of the respondents to make sure that respondents are not forced to either or opinion but rather allow them to choose to be neutral if they wish so.

### Household questionnaire

The survey collected the respondents’ socio-demographic characteristics and quantified women’s cancer knowledge levels. It also consisted of sections whose aim was to deduce the attitudes and practices of the respondents (Annex 1). Stratified random cluster sampling was employed in the selection of survey households and then one woman, who fit the eligibility criteria, was randomly selected from each household.

### Sampling

**Fig. 1:**
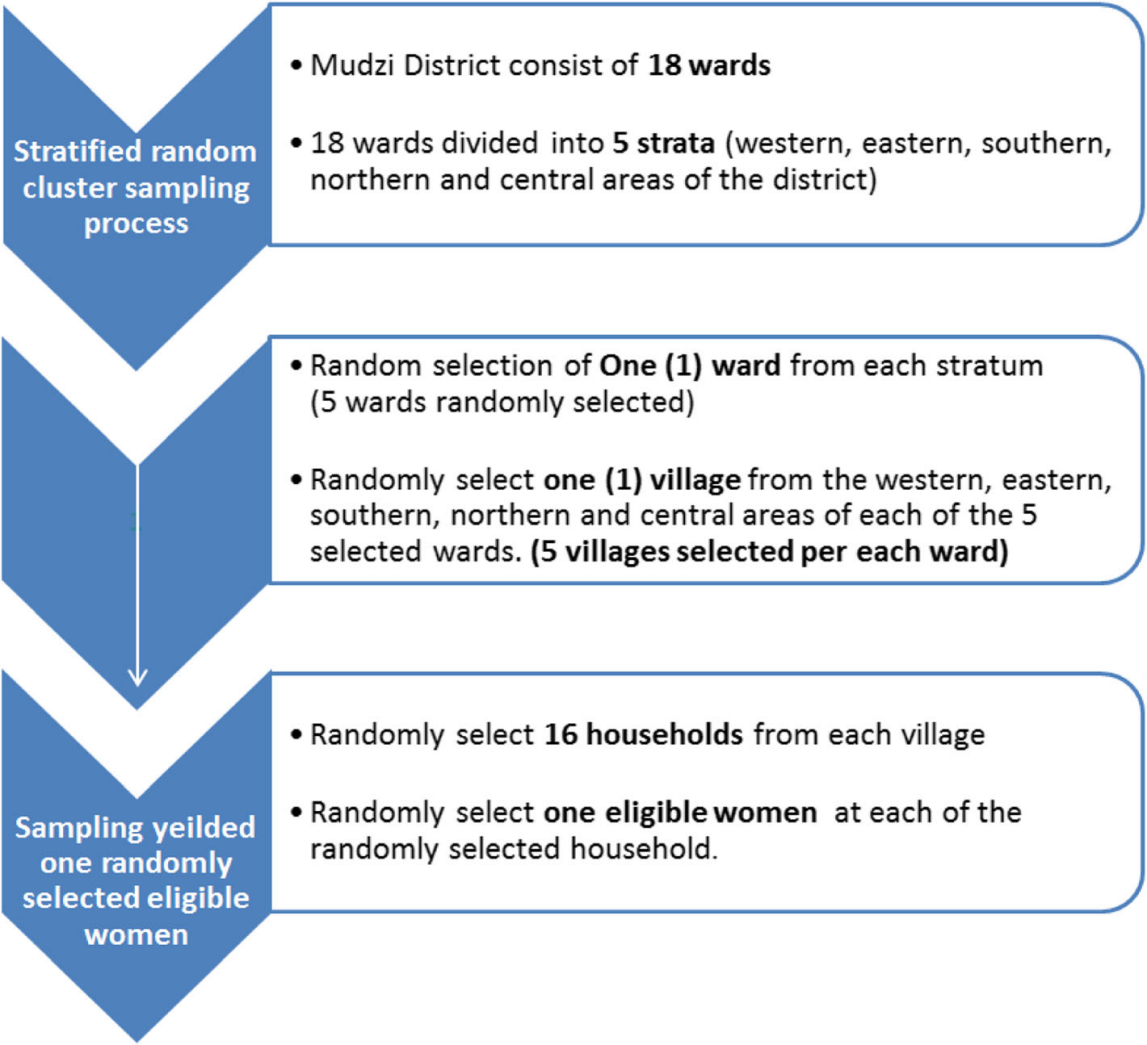
The stratified random cluster sampling process

The survey was conducted in five (5) out of eighteen (18) wards in Mudzi district. Stratified random cluster sampling was used in this survey. The first level was dividing the district into five strata namely northern, southern, western, eastern and central areas of the district. One (1) ward was then randomly selected from each stratum. This was followed by random selection of one village (second level) in each ward. At village level, households (third level) were randomly selected in line with the sample size. One woman aged 15–49 years was selected at each selected household. In households with more than one woman within the survey age range the enumerator would randomly select one woman from the household (Fig. 1). The advantages of stratified random cluster includes that each stratum/subgroup of the study population is considered unlike in simple random sampling method. This sampling method avoided potential bias inherent is simple random sampling by incorporating each stratum/cluster of the study population/area. Thus, all the 18 wards of the Mudzi district are well represented in the sample selection process.

### Ethical issues

The study received approval from the Medical Research Council of Zimbabwe (MRCZ) in 2014 (MRCZ/A/1823). The study was also approved by the Cancer Association of Zimbabwe board and the local rural authorities, including the district administrator (DA) and the district medical officer (DMO). The study respected each respondent’s freedom to participate and adhered to all research principles pertaining to privacy and confidentiality. Consent was sought from all the participants including parental consent and assent for the participants below 18 years old.

### Data analysis

Quantitative data were analysed using the Statistical Package for Social Scientists (SPSS), version 20 [13]. Data purification was done before analysis by checking data completeness, verifying random samples of the electronic data against the original data and running frequencies, means or ranges to detect errors and anomalous values. All errors and outliers were corrected by comparing with the original questionnaire. Univariate and bivariate analysis methods were used. Bivariate analysis was used to determine the relationship between knowledge, attitudes and practices, and the independent variables using Pearson’s chi-square test. The *P*-value of the chi-squared test was set at a 95% confidence interval.

## Results

### Socio-demographic characteristics of respondents

A total of 409 respondents were interviewed. The majority (72%) of the respondents were either married or cohabiting. Fifty-nine percent (59%) of respondents had a primary education and 37.4% had attained ordinary-level education. The majority (59.9%) of respondents belonged to the apostolic religion sect. (Table 1).

**Table 1:** Demographic and socio-economic characteristics of respondents (*N* = 409)

Demographic and Socio-economic variables		Response distribution	
		Frequency	%
Age Distribution (years)	15–19 years	76	18.6
	20–24 years	76	18.6
	25–29 years	63	15.4
	30–34 years	47	11.5
	35–39 years	66	16.1
	40–44 years	36	8.8
	45–49 years	45	11.0
	Total	409	100
Marital Status	Married/Cohabiting	294	72
	Never Married	57	14
	Divorced/Separated	29	7
	Widowed	29	7
	Total	409	100
Level of Education	None	11	2.7
	Adult literacy	3	0.7
	Primary	241	58.9
	Ordinary Level	153	37.3
	Diploma	1	0.2
	Degree and above	0	0
	Total	409	100
Religious Affiliation	Apostolic Sect	245	59.9
	Pentecostal	61	14.9
	Roman Catholic	20	4.9
	African Traditional Religion	7	1.7
	Protestant	39	9.5
	None	33	8.1
	Other	3	0.7
	Total	408	100
Employment Status	Formally employed	4	1
	Informally employed	61	15
	Student	25	6
	Not employed	319	78
	Total	409	100

### General cancer knowledge levels

Nearly 85% of respondents reported having heard about cancer. The most commonly-known cancers were cervical cancer (65.3), breast cancer (60.4%), Kaposi sarcoma (8.1%) and colon cancer (4.4%) (Tables 2 and 3).

**Table 2:** Respondents who have heard of cancer, by age group (N = 409)

			Heard about cancer		Total
			Yes	No	
Age of respondent	15–19 years	Count	49	27	76
		% within age of respondent	64.5%	35.5%	100.0%
	20–24 years	Count	65	11	76
		% within age of respondent	85.5%	14.5%	100.0%
	25–29 years	Count	54	9	63
		% within age of respondent	85.7%	14.3%	100.0%
	30–34 years	Count	41	6	47
		% within age of respondent	87.2%	12.8%	100.0%
	35–39 years	Count	61	5	66
		% within age of respondent	92.4%	7.6%	100.0%
	40–44 years	Count	34	2	36
		% within age of respondent	94.4%	5.6%	100.0%
	45–49 years	Count	41	4	45
		% within age of respondent	91.1%	8.9%	100.0%
Total		Count	345	64	409
		% within age of respondent	84.4%	15.6%	100.0%

**Table 3:** Respondents with knowledge of types of cancers (*N* = 409)

Types of cancers known^a^	Respondents reporting knowledge of the cancer types	
	*N*	%
Breast Cancer	247	60.4
Cervical Cancer	267	65.3
Bladder Cancer	1	0.2
Kaposi sarcoma	33	8.1
Colon and Rectal Cancer	18	4.4
Endometrial Cancer (Uterus lining)	7	1.7
Kidney (Renal Cell) Cancer	2	0.5
Leukaemia (White Blood Cells)	5	1.2
Lung Cancer	6	1.5
Melanoma/Skin Cancer	7	1.7
Non-Hodgkin Lymphoma	5	1.2
Pancreatic Cancer	0	0
Prostate Cancer	7	1.5
Thyroid Cancer	1	0.2

^a^ *There is no statistically significant relationship between age and knowledge of types of cancers*

### Knowledge on breast and cervical cancer symptoms, risk factors and prevention

Over 18% of respondents did not know of any breast cancer symptoms, while only 27.8% could identify “a lump” as one of the sign of breast cancer (Table 4). More than half of the respondents (51%) reported that they did not know the signs and symptoms of cervical cancer. Vaginal bleeding and foul smelling vaginal discharges were noted as some of the common symptoms of cervical cancer, by 11 and 13% of the respondents, respectively (Table 5). Of all respondents, 34.2% reported that they did not know of any cervical cancer risk factors. Of all respondents, 22.2% identified “insertion of herbs into the vagina” as one of the common risk factors for cervical cancer. More than a quarter of respondents (29.9%) reported that they were not aware of how cervical cancer could be prevented, and 36.2% reported that they did not know any breast cancer prevention methods (Table 6).

**Table 4:** Respondents with knowledge of breast cancer symptoms (*N* = 409)

Knowledge of breast cancer symptoms^a^	*n*	%
A lump or thickening in or near the breast or in the underarm that persists through the menstrual cycle	46	11.2
A mass or lump, which may feel as small as a pea	68	16.6
A change in the size, shape, or contour of the breast	45	11
A blood-stained or clear fluid discharge from the nipple	20	4.9
Redness of the skin on the breast or nipple	20	4.5
Other (specify)___________	30	7.3
Do not know	75	18.3

^a^ *There is no statistically significant relationship between age and knowledge of symptoms of breast cancer*

**Table 5:** Percentage of respondents with knowledge of cervical cancer symptoms (*N* = 409)

Symptoms	%
Vaginal bleeding	11
Vaginal foul smelling discharges	13
Back ache	8.3
Pain during and after sexual intercourse	6
Other (Specify)_____________	16
Do not know	51

**Table 6:** Respondents with knowledge of risk factors of cervical cancer, methods of prevention of cervical cancer and methods of prevention of breast cancer

Risk factors of cervical cancer (*N* = 409)	*n*	%
Having multiple sexual partners	54	13.2
Early onset of sexual activity	12	2.9
Sexually transmitted infections (STIs)	21	5.1
Tobacco use	4	1
Insertion of herbs	91	22.2
Dry sex	17	4.2
Other (specify)	26	6.4
Do not know	140	34.2
Methods of prevention for cervical cancer (*N* = 409)	*n*	%
Early treatment of STIs	14	3.4
Avoid multiple sexual partners	47	11.5
Avoid early sexual intercourse	12	2.9
Quit Tobacco use	4	1
Through vaccination of HPV vaccine	3	0.7
Practice safe sex	23	5.6
Avoid insertion of herbs/dry sex	82	20
Encourage partner to go for male circumcision	26	6.4
Regular screening	16	3.9
Other (Specify)	9	2.2
Do not know	122	29.8
*There is no statistically significant relationship between age and knowledge of preventing the development of cervical cancer*		
Prevention methods for breast cancer (*N* = 409)	*n*	%
Breast cancer screening(self-breast examination or mammogram)	65	15.9
Avoid exposure to environmental carcinogens	16	3.9
Reduce alcohol intake	0	0
Quit smoking	3	0.7
Exercising regularly	249	60.9
Balanced diet	6	1.5
Other (Specify)_______________	15	3.7
Do not know	148	36.2
*There is no statistically significant relationship between age and knowledge of methods of preventing breast cancer.*		

Knowledge levels of breast cancer risk factors are low. Forty-six percent of respondents reported that they did not know of any breast cancer risk factor. Although tobacco consumption is a well-documented risk factor responsible for a third of the cancers worldwide [1], only 1% of the respondents identified tobacco-use as a risk factor. Eighty-one percent of the 27 respondents who knew “other” breast cancer risk factors cited “putting money in the bra” as a risk factor.

The study revealed low knowledge of cancer risk factors and ways of preventing breast and cervical cancers. Only 15.9% could identify breast self-examination and mammography as methods of preventing breast cancer. None of the respondents identified “reducing alcohol intake” as a way of preventing breast cancer. Only 2.9% of respondents identified avoiding early sexual intercourse, early treatment of sexually transmitted infections (3.4%), safer sex (5.6%), regular screening (3.9%), and vaccination (0.7%) as ways of preventing cervical cancer (Table 6).

### Attitudes on breast and cervical cancer

Fourteen percent (14%) of respondents strongly disagreed, 1.6% disagreed and 4.8% neither agreed nor disagreed with the statement that “any adult woman, including I, can develop breast and cervical cancer”. In addition, 17.5% agreed, 3.2% strongly agreed and 28% neither agreed nor disagreed with the statement that “cervical cancer is a disease for prostitutes”. Nineteen percent (19%) of the respondents strongly agreed that they would rather not know if they had cancer, and would prefer to stay ignorant of their cancer diagnosis. A small proportion (2.2%) strongly agreed, 10.9% agreed and 5.4% neither agreed nor disagreed with the statement that “Getting breast and cervical cancer is a death sentence” (Table 7).

**Table 7:** Respondent attitudes toward statements on breast and cervical cancer (N = 409)

Statement	Strongly agree (%)	Agree (%)	Neither agrees nor disagree (%)	Disagree (%)	Strongly disagree (%)
Any adult woman including me can develop breast or cervical cancer	23.2	56.1	4.8	1.6	14.3
Cervical cancer is a disease for prostitutes	3.2	17.5	13.4	28.0	37.9
Breast and cervical cancer are diseases for the elderly women	3.2	7.0	8.0	36.9	44.9
I would rather not know if I had breast or cervical cancer	18.5	2.5	1.9	34.1	43.0
Getting breast and cervical cancer is a death sentence. There is not much that can be done when someone has breast or cervical cancer	2.2	10.9	5.4	28.2	53.2
Talking to family/friends about symptoms of breast or cervical cancers is embarrassing	1.9	2.9	1.0	35.0	59.2

### Breast and cervical cancer practices

Approximately 55% of the respondents reported that they had never discussed cancer issues with their partners in the past 12 months and 27.4% had discussed cancer issues with their partners. Most of the respondents (96.2%) had never received cervical cancer screening. There was no statistically significant relationship between age and having undergone cervical cancer screening. The majority of the respondents (70.8%) had never discussed breast cancer issues with other community members. Similarly, approximately 70% reported that they had never discussed cervical cancer issues with community members (Table 8).

**Table 8:** Respondent attitudes toward statements related to breast and cervical cancer prevention practices (*N* = 409)

Statement	Response Category	Percent
Ever discussed general cancer issues with a partner or spouse in the last three months	Yes	27.3
	No	54.8
	Do not remember	0.3
	Not Applicable	17.6
	Total	100
Ever Screened of Cervical screening (VIAC or Pap smear test)	Yes	3.8
	No	96.2
	Total	100
Last time of having cervical cancer screening (for only the respondents who had had a cervical cancer screening test)	In the last 12 months	33
	In the last 2 years	17
	More than two years ago	50
	Total	100
Ever discussed cervical cancer related issues other community members	Yes	20.9
	No	70.2
	Don’t remember	1.1
	Total	100
Ever discussed cervical cancer related issues other community members	Yes	27.9
	No	70.8
	Don’t remember	1.3
	Total	100
Ever had Self Breast examination	Yes	50.3
	No	49.7
	Total	100

## Discussion

In order to achieve Sustainable Development Goal 3 [14] and World Cancer Declaration (WCD) 2013’s overarching goal of reducing premature deaths from cancer, improving quality of life and increasing cancer survival rates worldwide, [15] sub-Saharan African countries need to focus on community based approaches for both cancer information dissemination and access to cancer services. To help reach these goals, this study set out to assess rural Zimbabwean women’s knowledge, attitudes and practices relating to breast and cervical cancer.

The majority of respondents were aware of breast and cervical cancer as diseases, and some were also aware of Kaposi’s sarcoma (8.1%) and colon cancer (4.4%). Knowledge of different cancers seems to correlate with the prevalence of different cancers in the country, as the Zimbabwe National Cancer Registry [3] demonstrates that breast and cervical cancer contributed the most to Zimbabwe’s overall cancer burden in 2015, followed by Kaposi’s sarcoma and colon cancer [3].

Nearly two-fifths of respondents were not aware of breast and cervical cancers at all, significantly lower than respondents in similar studies elsewhere in Africa [7–10]. Moreover, the majority of the respondents in Chipfuwa, et al.’s study (84.4%) in urban Zimbabwe had heard about cancer [6]. Still, the rate of knowledge was much higher than in our present study in rural Zimbabwe, suggesting that there is a strong urban-rural divide in cancer knowledge in Zimbabwe. Therefore, relevant stakeholders must further disseminate cancer-related information in rural Zimbabwe to bridge this apparent gap.

A similar study in sub-Saharan African countries by Perlman, et al. showed that study participants were willing to accept the HPV vaccine, but that they had low levels of knowledge and awareness of cervical cancer in general [16]. Similarly, study findings in Bindura, Zimbabwe showed that the majority of the respondents (69.4%) did not know the risk factors of breast cancer [6]. These results show that while targeted action is required in rural Zimbabwe, the rest of the country, and the region in general, also require cancer knowledge dissemination.

Healthcare providers are generally considered to be custodians of correct health information. However, the present study revealed that the most common sources of cancer information were television and radio (40.8%) and that health workers were identified as cancer information sources by only 15.6% of the respondents. This finding is in contradiction to the one demonstrated by Chipfuwa, et al. In their Bindura, Zimbabwe study, health care providers were shown to be the most often cited source of cancer-related information (30%). Friends (18.1%) and the radio (11.2%) were both much less likely to be cited [6]. The respondents in the Bindura study, unlike our Mudzi study, consisted largely (84.9%) of urban residents.

These results related to healthcare providers may point to a necessity to further train Zimbabwean rural health workers. In a study among university students in Angola, knowledge on breast cancer symptoms was found to be low among both medical and non-medical students. Fewer than 40% of the participants knew that changes in colour or shape of the nipple could be a sign of breast cancer [17]. A regional study in East, Central and Southern Africa showed that health workers believed themselves to be at low risk of developing cervical cancer [18]. Such a perception results in delayed medical attention as evidenced by the fact that 81% of new cancer cases in Zimbabwe are diagnosed late [3]. In the context of this regional data and the findings of the present study, the capabilities of rural health workers in Zimbabwe need to be improved.

HIV and AIDS augment the rate of HIV-related cancers as 60% of Zimbabwe’s new cancers are associated with it [10]. Zimbabwe’s HIV and AIDS prevalence rate among the 15–49 age group is 13.8% [4]. Prevalence is higher among women (16.7%) than among men (10.5%) [4]. Despite the prevalence of HIV and its associated risk factors, nearly two-fifths of respondents reported having no knowledge of the link between cervical cancer and HIV/AIDS. Twenty-two percent identified “insertion of herbs into the vagina” as one of the common possible risk factors of cervical cancer while 34.2% of respondents reported not knowing any cervical cancer risk factor. Insertion of herbs into the vagina for perceived improved sexual pleasure is a common practice in Mudzi; however, no study has been done to determine the relationship of this traditional practice and cervical cancer.

In Zimbabwe, Visual Inspection with Acetic Acid and Cervicography (VIAC) is recommended as a cervical cancer screening modality [19]. However, this study showed that rural women generally did not recognise regular screening as a key method of preventing cervical cancer. Although the structure of the health system in Zimbabwe is such that it is decentralised to ward level, the capacity of the peripheral rural health centres to provide both correct cancer information/education and basic breast and cervical cancer screening is minimal.

The study revealed low self-risk perception and ignorance about cancer diagnosis. The low self-risk perception agrees well with Chipfuwa, et al.’s 2014 studies in Bindura, Zimbabwe, in which the authors found that 53% of the respondents did not think that they could develop breast cancer [6]. Mudzi health staff study revealed that 73.3% believed that they were not at risk of developing cervical cancer [12].

Cervical cancer screening is highly recommended by the World Health Organization (WHO) to prevent invasive cervical cancer [1, 20]. As a consequence, the government of Zimbabwe’s screening guidelines recommend cervical cancer screenings for every three years for all women, and every year for HIV positive women [5, 21]. However, most of the respondents in this study (96.2%), had never received the screening. In this study, 49.7% of respondents had never done breast self-examination. Similarly, findings by Chipfuwa, et al. revealed that only 20.9% of the respondents had undergone a mammography [6].

Review-level evidence suggests that reasons for low breast and cervical cancer screening uptake among women in sub-Saharan Africa are generally similar, despite the enormous diversity of the region. Women throughout the continent have reported fear of screening procedure and negative outcome, low level of awareness of services, embarrassment and possible violation of privacy, lack of spousal support, societal stigmatisation, cost of accessing services and health service factors like proximity to facility, facility navigation, waiting time and health care personnel attitude [22].

Results show low community dialogue regarding cancer, as approximately 70% reported that they had never discussed breast and cervical cancer issues with community members. Community dialogue on cancer is low and this may lead to stigma and discrimination. Interventional studies need to be done to investigate current strategies to enable improvement in cancer knowledge, attitudes and practices, especially in rural areas.

The results from this study must be interpreted with some limitations. Though post-menopausal women also develop cancer, this study focused only on women of reproductive age. Moreover, this study was conducted in only five/18 wards of one district. It is therefore suggested that similar studies be conducted at a national scale, and with a broader target population. Analysis of the results did not account for the survey’s complex sampling design and results are only generalizable to the study population.

This cross-sectional study does not evaluate the cancer intervention measures implemented in Zimbabwe. Interventional studies should be carried out to measure the effectiveness of the current intervention measures in order determine cost effective, sustainable and evidence-based interventions. Further research should be carried out on breast and cervical cancer tertiary prevention and quality of life of cervical cancer survivors which are two severely under-researched areas in Zimbabwe and sub-Saharan Africa in general [22].

## Conclusion

Breast and cervical cancers account for almost half of all new cancer cases among women in Zimbabwe. The general lack of awareness and comprehensive knowledge about breast and cervical cancer noted in this study revealed the gaps currently existing in the national cancer prevention and control programme.

Low self-risk perception, low uptake of cancer early detection services and lack of capacity of local health centres to provide cancer services need immediate attention. Therefore, scaling-up cancer information dissemination and early detection services must be prioritized. This scaling-up must include capacitation of local health institutions, in order to reduce both breast and cervical cancer morbidity and mortality. Both World Cancer Declaration target 5 (reducing stigma, damaging myths and misconception) and target 6 (universal screening, early detection) can only be achieved if rural communities have correct information about cancer and accessible early detection services.

## Abbreviations

AIDS: Acquired Immune deficiency syndrome
DA: District Administrator
DMO: District Medical Officer
HIV: Human Immuno- Virus
HPV: human papilloma virus
MoHCC: Ministry of Health and Child Care
MRCZ: Medical Research Council of Zimbabwe
SSA: Sub-Saharan Africa
UICC: Union for International Cancer control
VIAC: Visual Inspection with Acetic Acid and Cervicography
WCD: World Cancer Declaration
WCR: World Cancer Report
ZDHS: Zimbabwe Demographic and Health Survey
